# Gunshot Wound

**Published:** 1854-01

**Authors:** S. O. Long


*Gunshot Wound.* By S. O. Long, M. D.

I was called on the night of April 25, 1852, to visit Mr. H———, some four miles distant, who was shot about an hour previously with a double-barrel gun, five balls taking effect. Present condition: He was covered with blood, very much prostrated, in excruciating pain, perfectly calm, but not expecting to survive the injury he had received. In examining the wounds, I found one ball had struck him on the radial side of the fore-arm, cutting out one of the initials of his name. Another passed through his clothes, abrading the skin several inches on his breast, just below the nipple. The third passed backwards through the superficial muscles of the neck, in the posterior triangle. The fourth about an inch above, through similar parts, and fifth and last ball struck the ramus of the jaw anterior to the ear. Not finding an outlet for this ball, I passed a probe in the orifice extended it under the concha, around the base of the skull to the ligamentum-nuchæ, where I found and extracted the same, by making an incision, which afforded him much relief. The concha and helix were much bruised, as if from the wadding. The hemorrhage which propably proceeded from division of the occipital and posterior auricular arteries, had subsided. Dressed the ear with lint dipped in blood, applied compresses over the track of the balls, also lint moistened with oil, over the ball holes; directed Morphine grs. 2, water ℥ij. a tea-spoonful to be given every half hour, until pain and restlessness were entirely controlled, then every four hours. 26th, patient quiet and easy, directed the above solution as the symptoms required. 27th, patient complains of pain in the head and wounds, face flushed, skin hot and dry, tongue covered with white coat, pulse quick and frequent, urine scanty and high colored. Directed cold applications to the head Sul. mag. ℥i tart-emetic gr. ss., and repeat every three hours, until the bowels move freely, then give two tea-spoonfuls of the following mixture every two hours: Tart. Ant. 1 gr. Nitrate Potash ℥i, and apply the warm water dressing to the wounds. 28th, patient more comfortable, pulse natural, tongue moist and cleaning, surface cool. The sul. mag. et ant. acted freely on the bowels. Directed the ant. and potash every four hours. Appearance of wounds: The ball-hole on the ramus filled with reddish lymph, the track impervious, the incision united; the track of the balls in the neck changed to dark-red color: dressed with stimulating lotion. 30th, patient improving, omitted all medicines. The liquid matter in the orifice of the superior wound had organized. The wounds in the neck assumed a dark yellow color. Treatment the same. May 2d, removed the slough from the wounds on the neck. The wound on the jaw had healed over. Dressed with digestive oint., applied adhesive straps to prevent the wound from gaping, and bandage; directed a generous diet. 3d, wounds suppurated freely, pus laudable, treatment continued. 11th, patient doing well, the wound filled with healthy granulations. Directed the zinc lotion to restrain profuse suppuration. 13th, coated the surface with collodion, which produced a comfortably feeling, and support to the parts: dismissed the patient. June 17th, Mr. H. called and informed me that three weeks before, he had exerted himself in his store, and irritated the parts, and one of the wounds had opened, which presented exuberant pale granulations. I removed the same with the nitrate of silver, and dressed with simple cerate. It healed soundly, and he has since enjoyed good health, and has been engaged in active business.

